# Protocol for high-throughput compound screening using flow cytometry in THP-1 cells

**DOI:** 10.1016/j.xpro.2021.100400

**Published:** 2021-03-19

**Authors:** Stephan H. Spangenberg, Reza Beheshti Zavareh, Luke L. Lairson

**Affiliations:** 1 Department of Chemistry, The Scripps Research Institute, 10550 North Torrey Pines Road, La Jolla, CA 92037, USA

**Keywords:** Flow cytometry/mass cytometry, Cell-based assays, High-throughput screening, Immunology

## Abstract

Flow cytometry is a valuable method for analyzing protein expressions at the single cell level but can be difficult to apply to large numbers of samples. This protocol provides instructions to perform a high-throughput small molecule screen using flow cytometry analysis of THP-1 cells, a human monocytic leukemia cell line. We describe a methodology for identifying compounds that regulate PD-L1 surface expression in IFN-γ-stimulated cells, which has been successfully used to screen a collection of ∼200,000 compounds.

For complete details on the use and execution of this protocol, please refer to Zavareh et al. (2020).

## Highlights

• Protocol for high-throughput screening of compounds using flow cytometry
• Designed for quantification of cell surface expression of proteins in THP-1 cells
• This protocol has been used to identify modulators of PD-L1 expression

## Graphical Abstract

**Figure:**
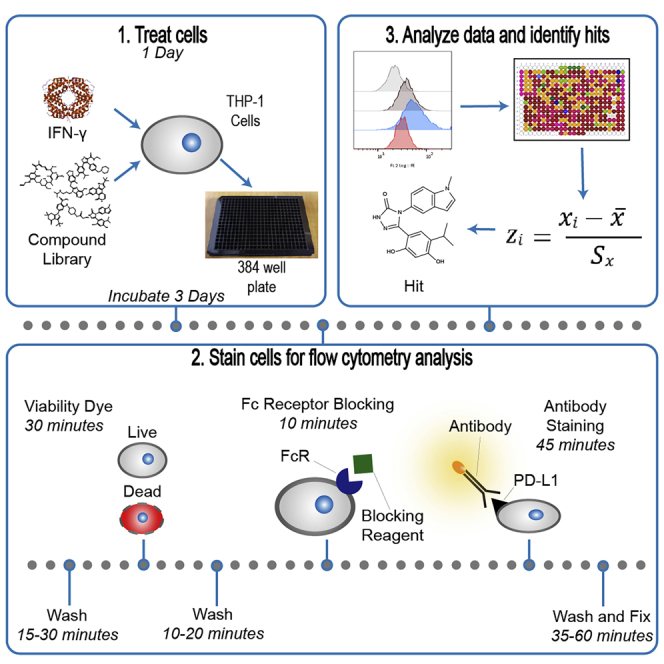

## Before you begin

### Experimental design

This protocol describes a high-throughput screening assay for the quantitative analysis of cell surface expression of PD-L1 on THP-1 cells. PD-L1 expression is induced by IFN-γ. This assay can be adapted to identify both activators and inhibitors of PD-L1 expression. Furthermore, this protocol can be adapted to analyze expression of other cell surface proteins, and to other cytokines or stimuli. This assay has been designed such that IFN-γ and test compounds are added to cells in rapid succession, and then incubated for three days. Compounds are added at the same time as IFN-γ because we have found this increases the likelihood of identifying compounds which have a small or moderate effect on PD-L1 expression. The three-day incubation time was chosen because we observed that this is approximately when cell surface PD-L1 expression peaks on THP-1 cells treated with IFN-γ. However, if this protocol is adapted to study other target proteins or other stimuli, the timing of compound addition and incubation will likely need to be optimized.

When designing a screen, the investigator must consider the number of compounds to screen and how many replicates to include. This protocol is ideal for testing about ten to twelve 384 well plates at a time. If two rows and two columns on each edge of the plate are excluded, to provide room for controls and to minimize edge effects, this allows for 240 test wells per plate, or about 2400 compounds per screen (without replicates). If compound library size and reagent costs allow, this assay should ideally be performed with three replicates of each plate. However, if a large number of compounds need to be screened, replicates can be omitted, but this incurs the risk of missing potential hits. In either case, all hits should be reanalyzed with three or more replicates and at several concentrations.

### Preparation of screening libraries

Compounds are prepared in 384 well compound plates (“source plates”). Compound libraries can be purchased directly from manufacturers or prepared in house. If screening plates are to be prepared in house, compounds should be dissolved in HPLC-grade DMSO or a similar solvent at a consistent, high concentration (e.g., 2 mM). Compound plates can include all relevant controls (such as vehicle only wells and reference compounds). Alternatively, controls can be added via dedicated control source plates. It is advisable to include compounds with known function as additional controls, if such compounds are available. For example, JAK Inhibitor I (Millipore Sigma) will inhibit IFN-γ signaling and strongly decrease IFN-γ induced PD-L1 expression. We typically use it at 500 nM final concentration. Including control compounds like this allows the investigator to ensure that compound transfer from source plates was completed as intended, and that the cells are responding to stimuli as expected. It also provides a reference point for hit compounds that alter PD-L1 expression or cell viability.

## Key resources table

**Table:** 

REAGENT or RESOURCE	SOURCE	IDENTIFIER
**Antibodies**		

CD274 (PD-L1, B7-H1) monoclonal antibody (MIH1), PE	Thermo Fisher Scientific	Cat#12-5983-42; RRID:AB_11042286

**Chemicals, peptides, and recombinant proteins**		

RPMI 1640 (ATCC modification)	Thermo Fisher Scientific	Cat#A1049101
Antibiotic-antimycotic (100X)	Thermo Fisher Scientific	Cat#15240112
Fetal bovine serum, qualified, heat inactivated, USDA-approved regions	Thermo Fisher Scientific	Cat#10438026
JAK inhibitor I	Millipore Sigma	Cat#420099; CAS: 457081-03-7
IFN-γ recombinant human protein	Thermo Fisher Scientific	Cat#PHC4033
DPBS, no calcium, no magnesium	Thermo Fisher Scientific	Cat#14190144
UltraPure 0.5M EDTA, pH 8.0	Thermo Fisher Scientific	Cat#15575020
32% Paraformaldehyde (formaldehyde) aqueous Solution	Electron Microscopy Services	Cat#15714-S

**Critical commercial assays**		

Fixable Viability Dye 660	Thermo Fisher Scientific	Cat#65-0864-14
FcR Blocking Reagent, human	Miltenyi Biotec	Cat#130-059-901

**Experimental models: Cell lines**		

Human: THP-1 cells	ATCC	TIB-202; RRID:CVCL_0006

**Software and algorithms**		

FlowJo	FlowJo LLC	RRID:SCR_008520; https://www.flowjo.com/solutions/flowjo/downloads
GraphPad Prism	GraphPad Prism	RRID:SCR_002798; https://www.graphpad.com/scientific-software/prism/
HyperView	IntelliCyt	https://intellicyt.com/products/software/

**Other**		

Cell Culture Microplate, 384 well, polystyrene, flat-bottom, μClear, black, tissue culture, sterile	Greiner Bio-One	Cat#781092
Compound Plate, 384 well, polypropylene, flat-bottom, 128/85 mm	Greiner Bio-One	Cat#781201-906
Cold Storage Adhesive Sealing Foil, Sterile	VWR	Cat#89049-034
Plate Orbital Shaker	Big Bear Automation	Cat#HT-91002
Biomek FX Automated Workstation	Beckman Coulter Life Sciences	N/A
BioTek ELx405 Plate Washer	BioTek	N/A
BioTek MicroFlo	BioTek	N/A
Multidrop Combi Reagent Dispenser	Thermo Fisher Scientific	Cat#5840300
CyAn ADP Flow Cytometer	Beckman Coulter Life Sciences	N/A
HyperCyt Autosampler	IntelliCyt	N/A

## Materials and equipment

### Preparing IFN-γ

IFN-γ should be aliquoted into single use aliquots and stored at −80°C. IFN-γ from Thermo Fisher (Cat#PHC4033) is supplied as a 1 mg/mL solution.

**Table:** THP-1 culture media

Reagent	Stock concentration	Final concentration	Amount
Heat-Inactivated Fetal Bovine Serum (FBS)	N/A	10% v/v	50 mL
Antibiotic-Antimycotic	100X	1X	5 mL
RPMI (ATCC Modification)	N/A	N/A	445 mL
**Total**		**N/A**	**500 mL**

Prepared THP-1 culture media should be stored at 4°C and should not be kept longer than two weeks.

**Table:** FACS buffer

Reagent	Stock concentration	Final concentration	Amount
Heat-Inactivated Fetal Bovine Serum (FBS)	N/A	2% v/v	10 mL
EDTA	500 mM	1 mM	1 mL
DPBS, no calcium, no Magnesium	N/A	N/A	489 mL
**Total**		**N/A**	**500 mL**

Prepared FACS buffer should be stored at 4°C and should not be kept longer than two weeks.

### BioMek FX pintool or other compound transfer system

We use the BioMek FX pintool to transfer 100 nl of compound from source plates to cell culture plates. Follow the manufacturer’s directions and safety procedures for using the BioMek FX. The following protocol is used to transfer compounds and then wash the pintool.

• Stamp compound plate.
• Stamp cell/assay plate, thereby transferring 100 nl of compound.
• Wash pintool with solvent and blot on filter paper three times using the following sequence of HPLC grade solvents.
  ○ DMSO
  ○ Isopropyl alcohol
  ○ Methanol
• After blotting the pintool with the last solvent wash of methanol, dry with fan. Replace filter paper frequently.

Any method of transferring compounds to a cell plate with sufficient throughput and precision may be used.

***Alternatives:*** Labcyte Echo with following modification to the protocol: compounds are spotted onto empty cell culture plates prior to adding cells.

### BioTek EL-405 or other plate washer

This plate washer is used to quickly and consistently aspirate supernatant from centrifuged plates in order to wash cells. The plate washer should be set to leave a thin layer of supernatant covering the bottom of the well to minimize cell loss and prevent cells from drying out between steps in the protocol. This will need to be optimized for individual plate washers and 384 well plates. We have set our EL-405 plate washer to aspirate to a height of 3.81 mm from the surface of the plate carrier, which leaves about 7 to 9 μl in the wells. It is important to clean plate washers thoroughly and frequently with water and/or ethanol to prevent clogs.

If a plate washer is unavailable, supernatants may be removed by flicking plates upside down over an appropriate receptacle. This should only be done after cells have been pelleted and only a single, quick, sure flick should be performed with each plate, to prevent cell loss. However, this flick method may lead to inconsistent volume left in wells, increased cell loss, and/or inconsistent results across different investigators.

**CRITICAL:** Be sure to follow best safety practices, institutional policies, and local regulations when handling or disposing of hazardous chemicals or biohazardous waste.

### BioTek MicroFlo Reagent Dispenser

A peristaltic pump-based reagent dispenser should be used to rapidly and precisely dispense reagents to plates.

***Alternatives:*** Thermo Fisher Multidrop Combi

### CyAn ADP flow cytometer and HyperCyt Autosampler

We used a Beckman Coulter ADP flow cytometer and a Hypercyt Autosampler for this screen. Any flow cytometer that can analyze 384 well plates may serve as an alternative.

***Alternatives:*** Sartorius iQue 3 or the BioRad ZE5 systems.

### Centrifuge

Use a refrigerated centrifuge capable of centrifuging multiple 384 well plates at the same time. If a centrifuge has a sufficiently deep bucket, multiple plates can be stacked. If plates are stacked, we recommend placing a lid on the top plate but removing lids from the rest of the plates. Since this protocol involves multiple centrifuge steps, the capacity of the centrifuge used can have a major impact on the throughput of the assay. If centrifuging large numbers of plates for the first time, we recommend testing the centrifuge with dummy plates filled with water or dispensable cells. By weighing plates before and after centrifuging, investigators can ensure that plates do not lose any content.

## Step-by-step method details

### Culture of THP-1 Cells

**Timing: 1–3 weeks**

In this protocol, THP-1 cells (Tsuchiya et al., 1980) are cultured until there are sufficient cells to perform the screen. Each full 384 well plate will require about 21 million cells. THP-1 cells are a suspension cell line and should not adhere to cell culture flasks when growing normally. Flasks should be kept static during cell growth, not shaken or otherwise agitated. Aseptic technique should be used to ensure the cells remain free of microbial contamination.

1. Culture THP-1 cells in suspension in T75 or T150 flasks and move to T225 flasks when 50 mL or greater of cell culture is achieved. Cells should be maintained between 300,000 and 800,000 cells per mL in THP-1 culture media. Adjust the frequency of passaging to ensure cells remain under 800,000 cells per mL, do not exhaust their media too quickly (as indicated by the media becoming acidified and the phenol red indicator turning yellow), and double about every three days. It is not necessary to use low attachment cell culture flasks.
2. Passage cells by diluting with media when cells approach 800,000 cells per mL.
  a. Mix cells well and take a sample to determine cell density. Dilute a sample of cell suspension in a 1:1 ratio with 0.4% Trypan Blue solution. Load 10 μl of cell suspension with Trypan Blue to a hemocytometer or automated cell counter slide and count cells.
  b. Dilute cells with fresh media to 300,000 cells per mL.
3. Passage cells with a complete media exchange approximately 3–4 days later (adjust timing of passaging to ensure proper cell density and prevent media exhaustion).
  a. Mix cells well and take a sample to count cells using 0.4% Trypan Blue and a hemocytometer or automated cell counter as described above.
  b. Centrifuge cells at 300 × *g* for 5 min.
  c. Resuspend in fresh media at 300,000 cells per mL.

**CRITICAL:** Do not allow cell concentration to exceed 1 million cells per mL under normal culture conditions.

### Titrate IFN-γ Activity

**Timing: 3–4 days**

IFN-γ should be titrated to determine the activity of a given lot of IFN-γ and THP-1 cells, and to determine the effective concentration at which 20% (EC20) and 50% (EC50) maximal PD-L1 expression is achieved. We recommend performing a dose response curve with at least 12 concentrations of IFN-γ and at least 10 replicate wells for each concentration. This can be adapted for other stimuli applied to cells.

4. Centrifuge THP-1 cells at 300 × *g* for 5 min. Resuspend cells in fresh media at 1.25 million viable cells per mL.
5. Distribute 40 μl of cell suspension per well to a 384 well plate using a reagent dispenser (e.g., the BioTek MicroFlo) or multichannel pipette.
6. Prepare dilution of IFN-γ.
  a. IFN-γ is prepared at 5X of its desired final concentration.
  b. A serial dilution should be performed. Start with IFN-γ diluted in THP-1 media at 10 μg/mL. Dilute by a factor of 2 to generate at least 12 concentrations (the final dilution should be about 4.9 ng/mL IFN-γ). Table 1 shows directions for performing an example serial dilution.

**Table 1:** Example serial dilution of IFN-γ

Dilution	IFN-γ (μL)	THP-1 media (μL)	[IFN-γ] (ng/mL) – 5X solution	[IFN-γ] (ng/mL) – Final concentration
1	4 (from 1 mg/mL stock)	396	10000	2000
2	200 (from dilution 1)	200	5000	1000
3	200 (from dilution 2)	200	2500	500
4	200 (from dilution 3)	200	1250	250
5	200 (from dilution 4)	200	625	125
6	200 (from dilution 5)	200	312.5	62.5
7	200 (from dilution 6)	200	156.25	31.25
8	200 (from dilution 7)	200	78.13	15.63
9	200 (from dilution 8)	200	39.06	7.81
10	200 (from dilution 9)	200	19.53	3.91
11	200 (from dilution 10)	200	9.77	1.95
12	200 (from dilution 11)	200	4.88	0.98
7. Transfer diluted IFN-γ to cell wells using a multichannel pipette. Transfer 10 μl per well, for a final well volume of 50 μl, and 50,000 cells per well. Each dilution should have at least 10 replicate wells.
8. Add 10 μl of media without IFN-γ to a set of 10 replicate wells to serve as an unstimulated control.
9. Incubate cells in a standard humidified 37°C 5% CO2 incubator for three days. Proceed with staining and flow cytometry analysis as described below beginning with step 21.
10. Graph the median fluorescence intensity of PD-L1 vs IFN-γ concentration in log. Generate a dose response curve and calculate the EC20 and EC50 for IFN-γ using GraphPad Prism or a similar program. In GraphPad Prism, the analysis method “[Agonist] vs. response – Find ECanything” can be used to calculate the EC20.

***Note:*** We recommend performing the screen with cells treated with the calculated EC20 of IFN-γ because we have observed that this relatively low level of stimulation allows for identification of both activators and inhibitors of PD-L1 expression. It also allows for the identification of compounds which might only have a moderate effect on PD-L1 expression but still present interesting avenues for future research. Treatment with higher levels of IFN-γ might eclipse the effect of compounds which activate PD-L1 expression, because expression may “max out” at a certain point. However, if the investigator is only interested in inhibitors of PD-L1 expression (or a different protein for which this protocol might be adapted), it may be more relevant to treat cells with a higher level of cytokine stimulation, such as the EC50 or EC80. Calculations can easily be adjusted accordingly with the “Find ECanything” function in GraphPad prism.

### Stimulate cells with IFN-γ and treat with compounds

**Timing: 1 day**

The screen is initiated by stimulating THP-1 cells and treating with screening compounds. This assay is designed to detect compounds which decrease or increase PD-L1 expression.

11. If compound source plates are frozen, remove them from the freezer at least 2 h before the planned transfer of compounds to allow ample time for them to equilibrate to approximately 20°C.
  a. Ensure plates are well sealed with an aluminum seal when they are removed from and returned to cold storage.
  b. After compound source plates have equilibrated to approximately 20°C, mix them well by vortexing at a high speed with the HT-91002 microplate orbital shaker in order to ensure the compounds are well dissolved following thawing. Plates should be vortexed for at least 1 min, until compounds are completely dissolved.
  c. After the compound plates have been mixed, centrifuge them at approximately 20–25°C (a refrigerated centrifuge will cause DMSO in the source plates to freeze) for 10 min at 1000 × *g* in order to ensure no compound remains on the aluminum foil seals. This is critical to ensure there is no cross contamination between wells.
12. Mix THP-1 cells well. Count THP-1 cells with a hemocytometer and 0.4% Trypan Blue.
  a. Calculate the number of cells needed for the screen. You will need 21 million viable cells per full 384 well plate.
13. Measure out sufficient THP-1 cells for the screen. Centrifuge cells for 5 min at 300 × *g*.
14. Aspirate the supernatant and resuspend in fresh culture media at a concentration of 1 million cells per mL (21 mL per screening plate).
  a. Pipette 1 mL of resuspended cells per plate into a separate centrifuge tube. These cells will be kept as unstimulated controls.
15. Add IFN-γ to the main tube of cells to a concentration equivalent to the EC20 of activation as determined in the previous IFN-γ titration section. In our hands, this is frequently around 50 ng/mL. Mix well by inverting the tube or pipetting up and down 5 to 10 times.
16. Distribute IFN-γ stimulated cells to the first 23 columns of the black 384 well assay plates, 50 μl per well (50,000 cells per well). Distribute the unstimulated cells to column 24, also at 50 μl per well.

***Note:*** The placement of control wells within the plate can be altered as the investigator prefers.

**CRITICAL:** If a multidrop or other reagent dispenser is being used to distribute cells, about 5 mL of extra cell suspension should be prepared, at the same concentration of cells and IFN-γ. This is to account for the dead volume of the reagent dispenser tubing and loss of cells during priming of the tubing.

17. Carefully label or barcode cell plates so they are clearly matched to compound source plates.
18. Remove the aluminum foil seal from a compound plate. Use the BioMek FX to transfer 100 nl of compound from the compound source plate to the cell plate. Seal compound plate with a new aluminum foil seal.
19. Repeat step 18 for each assay plate and compound source plate pair.
20. Incubate cells at 37°C 5% CO_2_ for 3 days.

### Stain cells for flow cytometry analysis

**Timing: 1 day**

After cells have been incubated for 3 days with IFN-γ and compounds, they can be stained for flow cytometry analysis. All centrifuge steps are performed for 5 min at 300 × *g*, at 4°C, unless otherwise noted. All reagents should be kept at 4°C or on ice unless otherwise noted.

***Note:*** This protocol is intended to detect cell-surface expressed PD-L1 and as such does not require a permeabilization step. The detection of intracellular targets will require membrane permeabilization with reagents such as saponin or Triton X-100 and likely will require further optimization. Permeabilization should only be performed on cells which have already been fixed.

21. Remove vials of Fixable Viability Dye (Thermo fisher Scientific) 660 from the freezer to allow them to thaw and equilibrate to approximately 20°C. Do not place vials on ice, as this will prevent the DMSO solvent from thawing.
22. Centrifuge cell plates.
23. Aspirate supernatant with the plate washer, leaving a thin layer of supernatant covering the pelleted cells.
24. Wash cells by dispensing 80 μl cold (around 4°C) DPBS without calcium or magnesium per well, then centrifuge and aspirate supernatant. From this point onwards, the plates should be kept on ice or refrigerated as much as possible. (Troubleshooting 1)

***Note:*** Cells are initially washed with DPBS, not FACS buffer, as protein-free DPBS reduces the background staining of the viability dye.

***Note:*** Fixable viability dyes are available in several colors. We chose this color (660, detected in the APC channel on most flow cytometers) because it is excited by the red laser on our Cyan ADP flow cytometer, whereas the fluorophore for our PD-L1 antibody is excited by our blue laser. This minimizes the possibility of spectral overlap between the two fluorophores. However, other color viability dyes are acceptable and should be selected with consideration of your chosen flow cytometer’s optical configuration.

25. Repeat step 24.
26. While the cells are centrifuging, mix viability dye concentrate by vortexing. Briefly spin down the tube with a benchtop microcentrifuge. Ensure the dye has completely thawed. Dilute the viability dye 1:1000 in cold PBS. Prepare 16 mL per plate, plus an extra 5 mL to account for the dead volume of the reagent dispenser tubing. Viability dye dilutions should always be prepared fresh.
27. After the second wash cycle with DPBS (DPBS has been dispensed, cells have been centrifuged, and supernatant has been aspirated), dispense 40 μl diluted viability dye per well. Vortex each plate without a lid with the HT-91002 microplate orbital shaker at speed setting 700 (arbitrary units) for about 5 s to resuspend the cells. From this point onwards, plates should be protected from light. Incubate plates at 4°C, with gentle rocking or orbital shaking, in the dark, for 30 min.

***Note:*** If you are using the HT-91002 microplate orbital shaker, or an alternative shaker, for the first time, gradually increase the speed to find a setting where the plate can be shaken vigorously enough to resuspend the cells without spilling the contents of the plate. The investigator can ensure that cells are being thoroughly resuspended by looking at the plate with a standard light microscope.

***Optional:*** If you have not used the viability dye before, or if you are performing a flow cytometry assay with more than two colors, you can heat kill cells to create a positive control for viability dye staining. Consult the manufacturer’s instructions for more information.

28. Following the incubation, wash cells by dispensing 40 μl FACS buffer per well. Centrifuge.
29. Aspirate the supernatant with the plate washer. Wash cells again by dispensing 80 μl per well FACS buffer. Centrifuge.
30. While cells are centrifuging, dilute Human FcR Blocking Reagent 1:20 in FACS buffer. 15 μl per well or about 6 mL per plate is required. FcR Blocking Reagent dilutions should always be prepared fresh.

***Note:*** The FcR Blocking Reagent prevents non-specific binding of antibodies to Fc receptors. Fc receptors are expressed on a variety of immune cells.

31. After plates have been centrifuged, aspirate supernatant and dispense 15 μl per well diluted Human FcR Blocking Reagent. Vortex plates without lids using the plate shaker, at speed setting 700 for 5 to 10 s each.
32. Incubate plates at 4°C, with gentle rocking or orbital shaking, in the dark, for 10 min.
33. During this incubation, prepare antibody dilutions. The PE conjugated antibody against human PD-L1, should be diluted 1:60 in FACS buffer (for an ultimate dilution of approximately 1:150). Enough volume for 15 μl per well (about 6 mL per plate with dead volume) is required. Antibody dilutions should always be prepared fresh.

***Note:*** We have used an anti-PD-L1 antibody conjugated to phycoerythrin (PE) because PE is one of the brightest fluorophores available for flow cytometry. Brighter fluorophores may make it easier to distinguish antibody staining signal from background fluorescence. However, any other fluorophore which has minimal spectral overlap with the viability dye should be acceptable. Always confirm that a given fluorophore is compatible with your flow cytometer before selecting it.

34. Following the 10 min incubation, without centrifuging the plates, dispense 15 μl per well of diluted antibody. We recommend leaving some wells unstained as a control. Vortex plates without lids using the plate shaker, at speed setting 700 for 5 to 10 s each.

***Note:*** The FcR Blocking Reagent does not need to be removed before adding the antibody solution.

35. Incubate plates at 4°C, with gentle rocking or orbital shaking, in the dark, for 45 min.
36. Following the 45 min incubation, dispense 40 μl per well FACS buffer. Centrifuge plates.
37. Aspirate supernatant, dispense 80 μl per well FACS buffer. Centrifuge plates.
38. Aspirate supernatant, vortex plates without lids using the plate shaker, at speed setting 700 for 5 to 10 s each.
39. Dispense 40 μl per well 4% paraformaldehyde (PFA) (Electron microscopy Services), diluted from the 32% stock in DPBS. 4% PFA solution should be prepared fresh.

**CRITICAL:** PFA is hazardous and should be handled in a fume hood using appropriate PPE and disposed of properly.

40. Incubate for 15 min at approximately 20°C, with gentle rocking or orbital shaking, with plates protected from light.
41. Dispense 40 μl per well FACS buffer. Centrifuge plates, aspirate supernatant, and vortex plates to resuspend cells. PFA waste should be collected and handled as hazardous chemical waste and disposed of according to local regulations and institutional policies.
42. Dispense 50 μl per well FACS buffer. Vortex plates again to ensure cells are resuspended and well mixed. The plates are now ready to analyze.

**Pause point:** Stained, fixed plates can be stored at 4°C, in the dark before flow cytometry analysis for 1 week.

### Flow cytometry analysis

**Timing: 1–2 h per plate**

This protocol describes flow cytometry analysis using a Beckman Coulter ADP flow cytometer with a HyperCyt auto-sampler.

43. Set up flow cytometer for analysis according to the manufacturer’s instructions. Ensure the exact same conditions and settings are used for each plate.
44. Before analyzing plates en masse, analyze a few control wells to ensure the flow cytometer is working properly.
45. Set gates:
  a. FSC-A vs. SSC-A to identify cells and eliminate debris
  b. FSC-H vs. FSC-A or FSC-H vs. Pulse Width to eliminate doublets
  c. APC-A vs. FSC-A to gate on viable cells. Fixable viability dye 660 is detected in the APC channel. Viable cells will have less staining with the fixable viability dye.
  d. PE-A vs. FSC-A, or PE-A histogram to determine PD-L1+ cells. Use no IFN-γ and/or IFN-γ + vehicle control wells to determine gates for PD-L1 high or low expression.
46. Analyze plates. The exact parameters of the analysis may need to be optimized by the investigator, especially if a different flow cytometer is used. We used the following protocol with the HyperView software and HyperCyt autosampler. Using this system, all wells in one plate are initially acquired continuously and data are stored as a single FCS file. (Troubleshooting 2)
  a. Pump speed: 15 rpm
  b. Prime: 60 s with sheath fluid
  c. Pre-shake: 15 s 3500 rpm
  d. Sample: 7 s
  e. Up time: 3 s
  f. Probe rinse: After every 12 wells, 1 s with FACS clean solution, 1 s with water
  g. Inter-well shake: After every 12 wells, shake 4 s 3500
  h. Post run flush: 60 s, sheath fluid.

***Note:*** The HyperCyt samples wells for the set “Sample time” and then takes up a bubble of air set by the “Up time”. This air bubble allows the HyperView software and the investigator to identify different wells when FSC is plotted against time. Refer to the HyperCyt and HyperView manufacturer’s instructions for more details.

47. Use the Well Identification function in the HyperView software to identify individual wells. Analysis can be performed in HyperView, or FCS files for each well can be exported for analysis in other flow cytometry analysis software such as FlowJo.

## Expected outcomes

**Figure 1:**
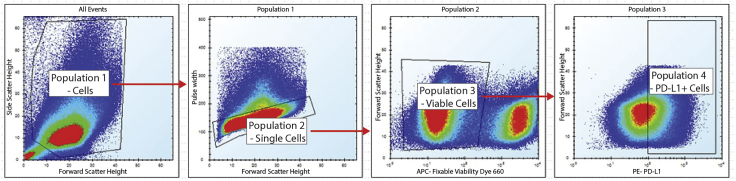
Gating strategy for flow cytometry data from a THP-1 screening plate This figure shows how HyperView can be used to set gates and enable analysis of one 384 well plate’s data. THP-1 cells were treated with compounds, stained, and analyzed by flow cytometry as described in this protocol. The first panel shows all events for every well of one plate, with a gate (population 1) for cells drawn. The second panel shows population 1, with a gate for singlets (population 2). The third panel shows population 2 with a gate for viable cells (population 3). The fourth panel shows population 3 with a gate (population 4) for PD-L1 positive cells, determined by examining individual control wells.

Following flow cytometry analysis, the investigator should be able to visualize the data in a fashion similar to the pseudocolored plots shown in Figure 1. These plots were generated in HyperView and show all events captured for every well of a 384 well plate. The first plot compares forward and side scatter height. Debris in the bottom left corner is gated out, and intact cells are designated population 1. In the second plot, forward scatter height and pulse width is compared. Doublets, identifiable by their pulse width being disproportionate to their forward scatter signal, are gated out. The third plot compares the APC channel—fixable viability dye—to forward scatter. The population with the lower staining intensity is the viable cells. Finally, the fourth plot displays PE intensity, which corresponds to staining with the PD-L1 antibody. The population 4 gate is designated PD-L1 high cells. This gate was set by looking at reference/control wells for no IFN-γ and IFN-γ plus vehicle control conditions.

**Figure 2:**
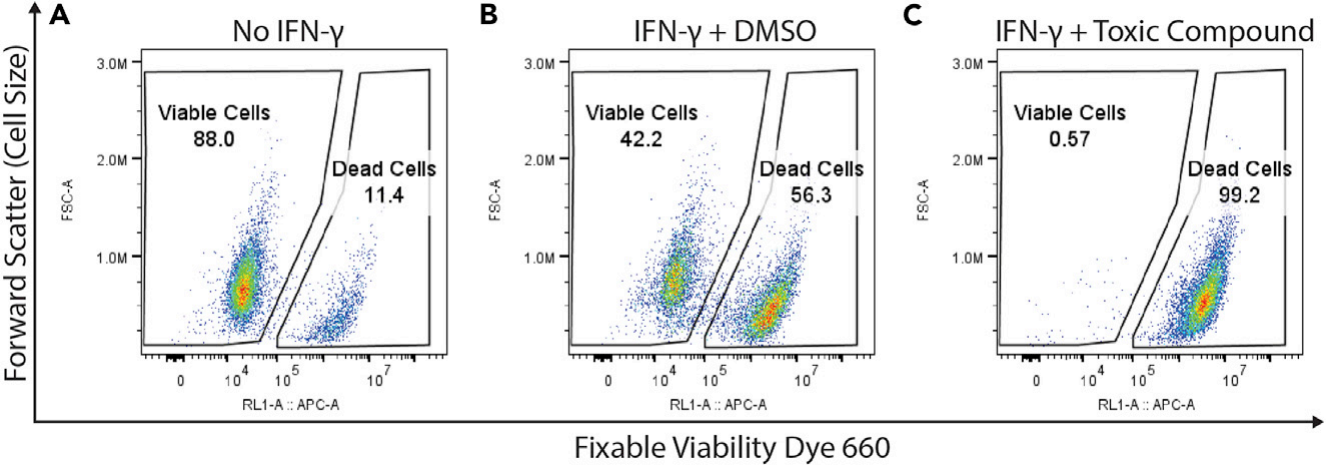
Example flow cytometry plots demonstrating gating for viable cells THP-1 cells were (A) untreated, (B) treated with IFN-γ (50 ng/mL, approximate EC20 of PD-L1 activation), or (C) treated with IFN-γ and a cytotoxic compound. Cells were then stained and analyzed by flow cytometer as described in this protocol. Plots show cells gated on singlets, as shown in Figure 1. Plots were generated with FlowJo. The numerical values under the name of each gate is the percent of parent for that gate.

**Figure 3:**
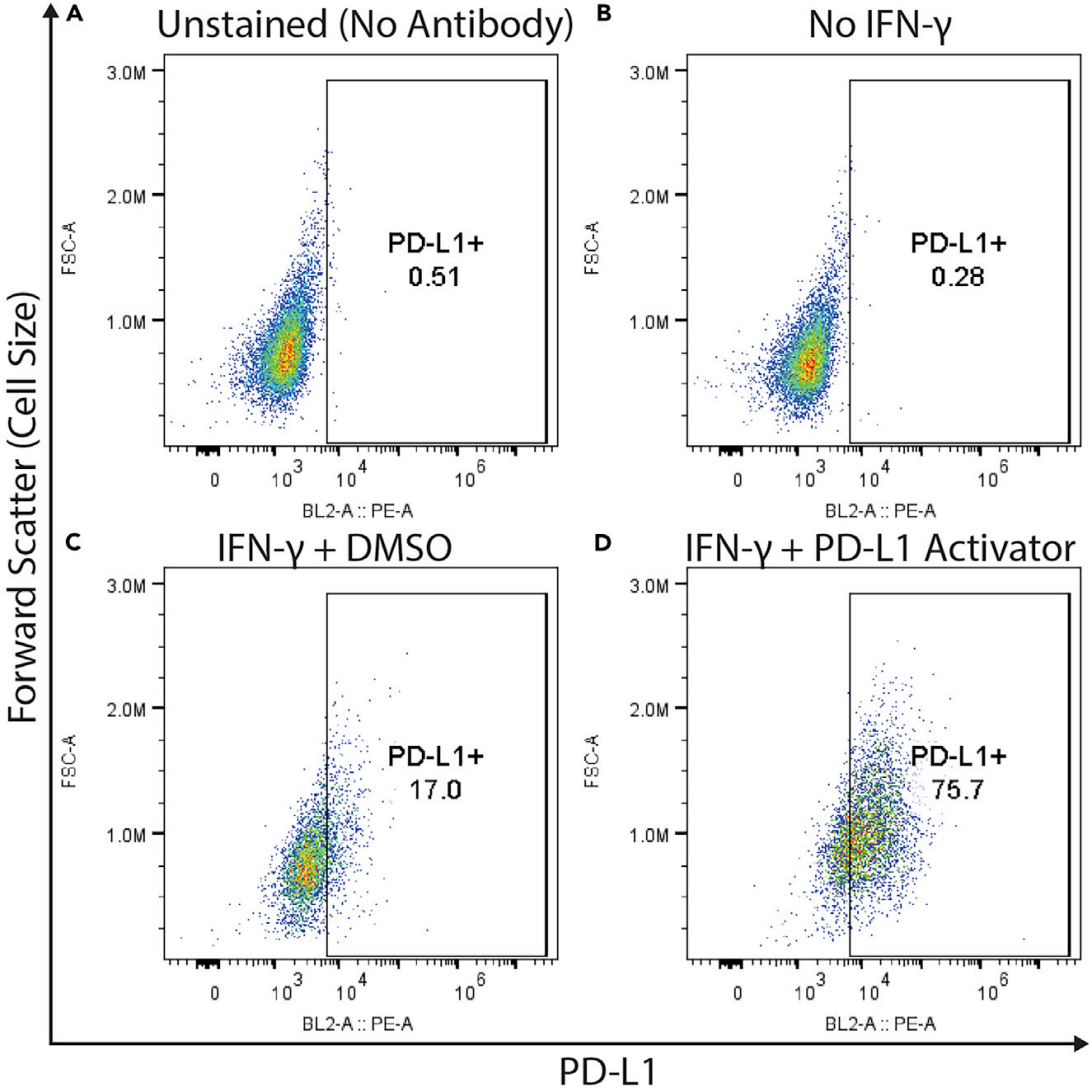
Example flow cytometry plots demonstrating gating for PD-L1 positive cells THP-1 cells were (A and C) treated with IFN-γ (50 ng/mL, approximate EC20 of PD-L1 activation), (B) left untreated, or (D) treated with IFN-γ and a compound which we have identified that increases PD-L1 expression (unpublished data). Cells represented in (A) were not stained with the PE conjugated anti-PD-L1 antibody, whereas (B, C and D) were. All four groups of cells were otherwise stained (including fixable viability dye) and analyzed by flow cytometry as described in this protocol. All plots depict viable cells, gated as seen in Figure 2.

Figures 2 and 3 demonstrate how gates for viable cells and PD-L1 positive cells are determined. Figure 2 shows representative flow cytometry plots of untreated THP-1 cells (Figure 2A), cells treated with IFN-γ alone (Figure 2B), or IFN- γ and a highly cytotoxic compound identified during a screen (figure 2C). The x-axis is fluorescence intensity for the Fixable Viability Dye 660 and the y-axis is forward scatter, which correlates with cell size. A more highly stained population of dead cells can be seen in each condition, with nearly all cells killed by the cytotoxic compound (Figure 2C). Figure 3 shows PD-L1 expression in viable cells. The gate for PD-L1 positive cells is positioned just above the max intensity of the population of no IFN-γ cells (Figure 3B). Stated differently, PD-L1+ cells are defined as cells with higher PD-L1 signal than the main population of no IFN-γ cells. Note that the unstained cells (Figure 3A) (which received no antibody) have similar fluorescent intensity to the no IFN-γ cells (Figure 3B) (which were stained with the anti-PD-L1 antibody). This suggests that this antibody and staining protocol produced very little non-specific binding or background fluorescence, and that PD-L1 is essentially not expressed at all in unstimulated THP-1 cells. The IFN-γ and DMSO cells (Figure 3C) have about 17% PD-L1+ cells, because they were treated with a concentration of IFN-γ equivalent to the EC20 of PD-L1 activation, as determined using this protocol (see steps 4–10). A compound identified using this screening protocol (Figure 3D) significantly increases PD-L1 expression.

**Figure 4:**
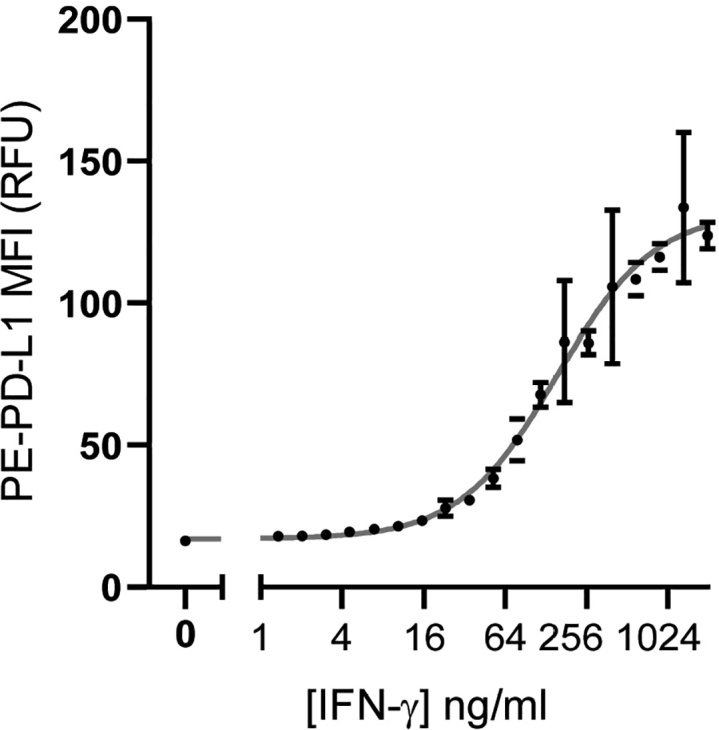
Example IFN-γ titration results IFN-γ concentration (on a log2 scale) is shown compared to PD-L1 signal (PE channel). Each point shows the mean of 10 replicate wells. Error bars show the standard error of the mean. The gray curve is the non-linear fit for the data. The calculated EC20 was 49.04. R-squared for the non-linear fit was 0.9397.

Figure 4 shows representative results from an IFN-γ titration. The concentration of IFN-γ is plotted against the median fluorescence intensity (MFI) of PE fluorescence (corresponds to PD-L1 expression). In this titration, the EC20 of PD-L1 activation was calculated to be 49.04 ng/mL. This calculation was performed using the function “[Agonist] vs. response – Find ECanything” in GraphPad Prism.

**Figure 5:**
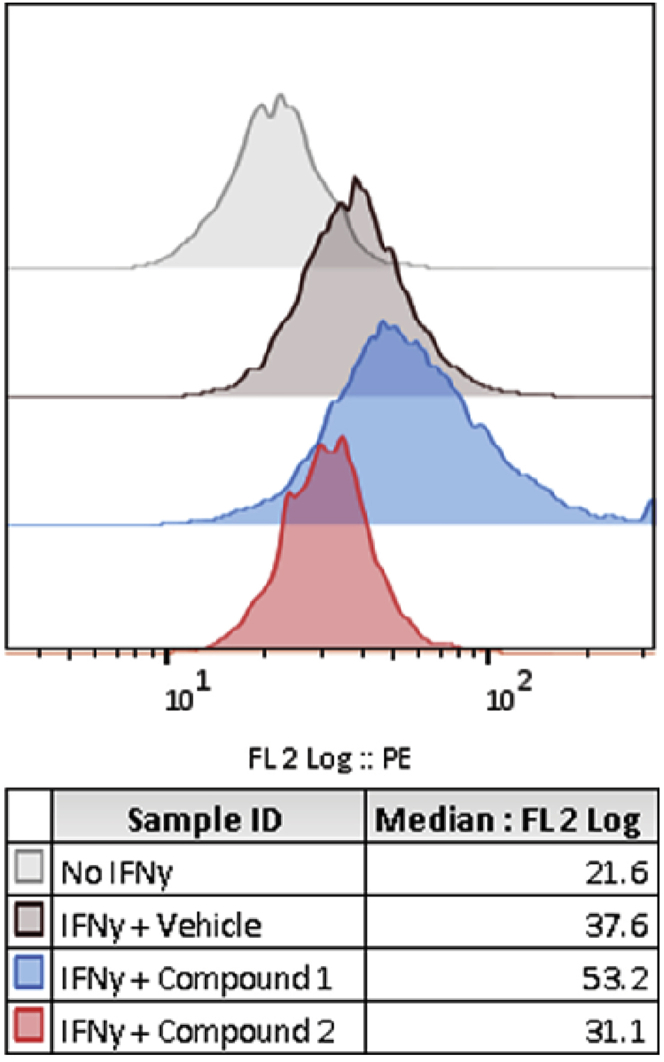
Representative control and compound treated wells Flow cytometry histogram plots showing PD-L1 expression (PE fluorescence signal) for wells treated with either No IFN-γ, IFN-γ and vehicle, or IFN-γ and one of two compounds from a screening library which were identified as modulating PD-L1 expression.

Figure 5 is a histogram showing the cell-based distribution of PE fluorescence intensity (corresponds to PD-L1 expression) for representative wells. Compound 1 increases PD-L1 expression compared to the vehicle control, whereas Compound 2 decreased PD-L1 expression.

Screening results can be tabulated and/or visualized as plate heat maps. Important parameters to examine include the median fluorescence intensity of your fluorescence channel of interest (PE in this example), the percent viable cells, and the percent PD-L1 high or low cells. Keep in mind that fluorescence intensity values use arbitrary units, and their exact values can vary from experiment to experiment even when the same conditions are used. Therefore, it is inadvisable to compare results from different flow cytometry runs unless a normalization method is used, such as Z-score (see quantification and statistical analysis).

The investigator should define relevant analysis parameters and screening hit cut-offs based on their own objectives, assay details, and screening libraries employed.

**Figure 6:**
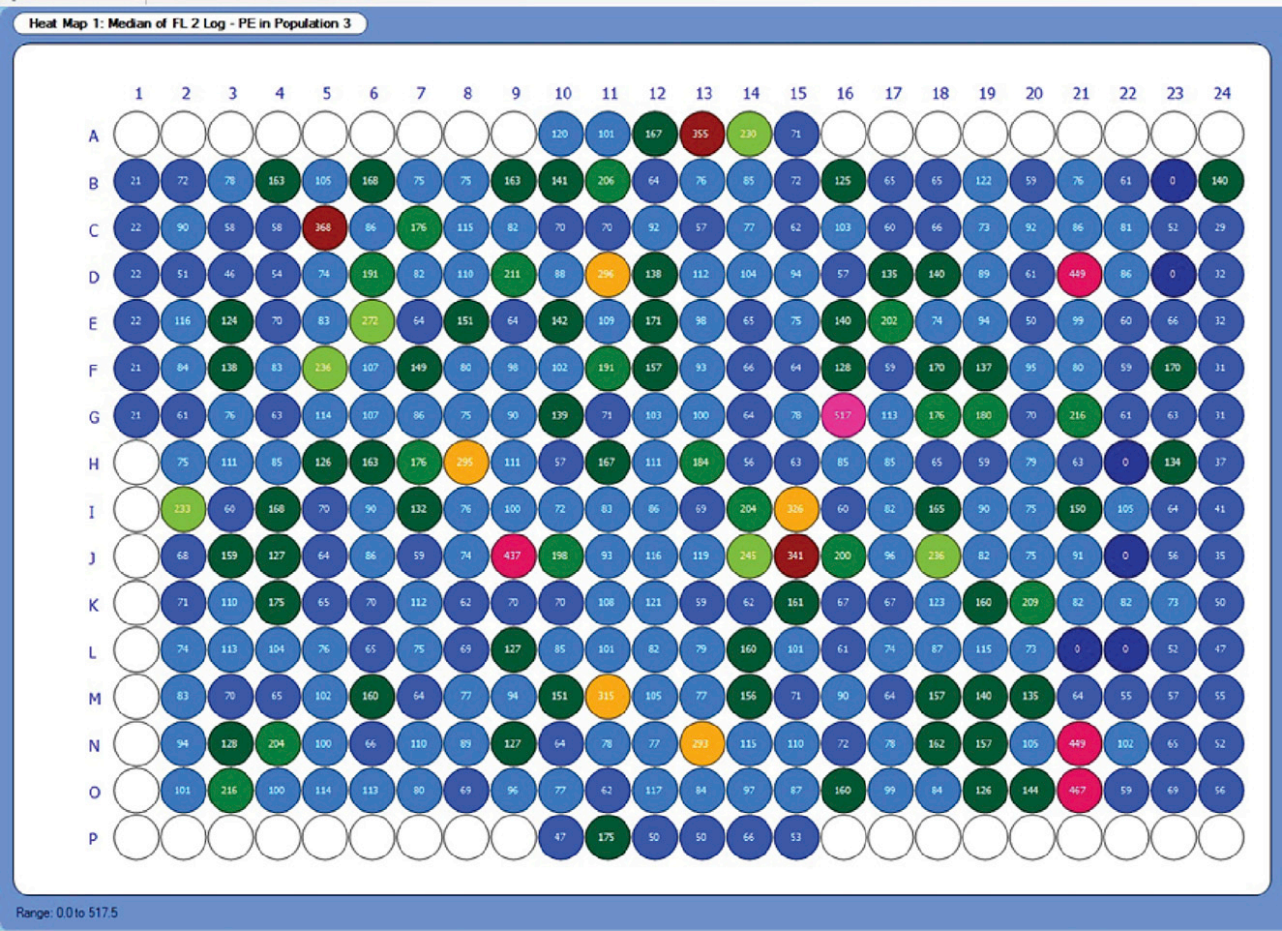
Heatmap of PD-L1 expression in a screening plate Heatmap, generated in HyperView, shows PD-L1 expression as the MFI of PE. Results are shown for population 3, which is viable cells.

**Figure 7:**
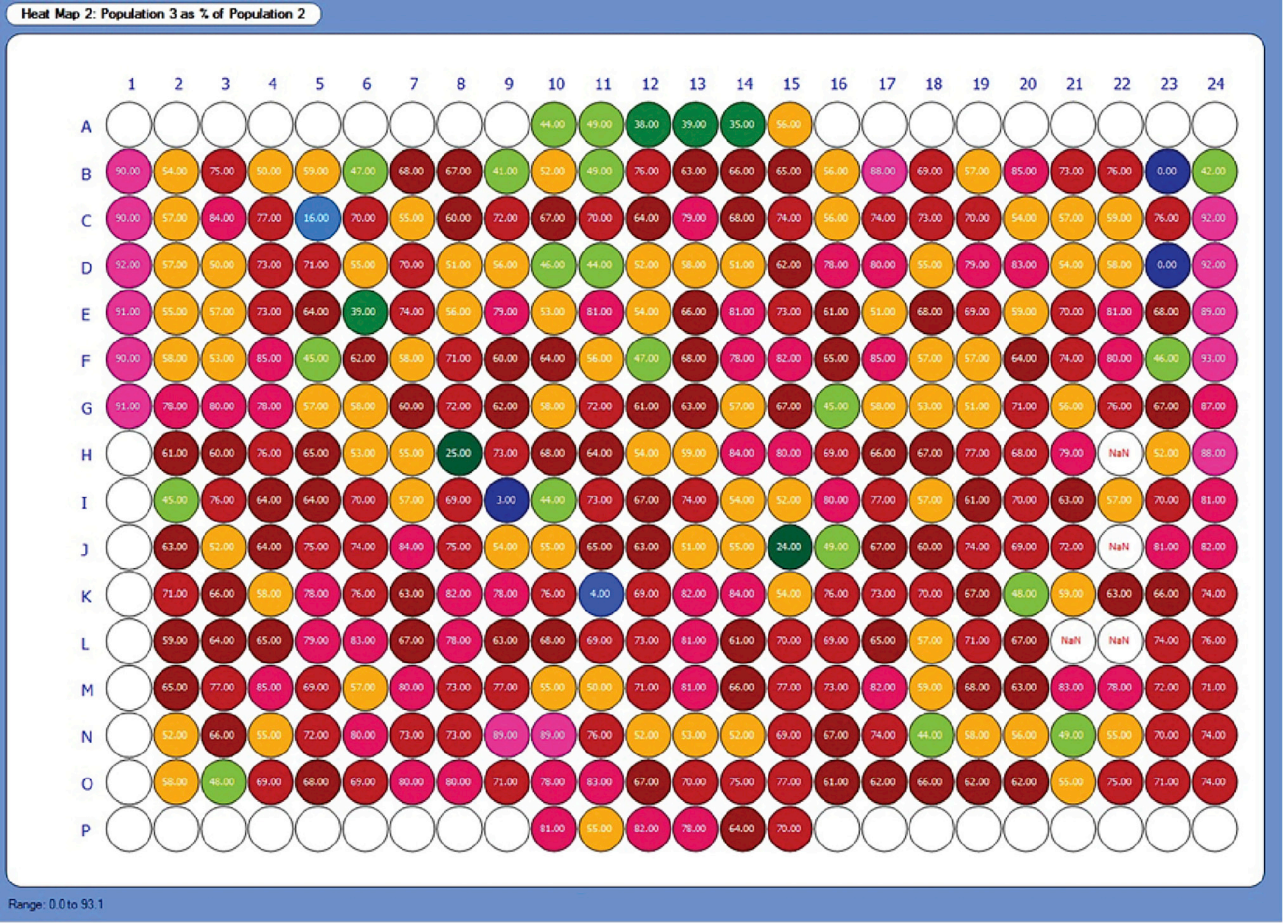
Heatmap of viability in a screening plate Heatmap, generated in HyperView, shows the percent viable cells for each well.

The example heat map in Figure 6 shows the median fluorescence intensity of PE (PD-L1 antibody) in each well, gated on live cells. The wells in column 1 were the no IFN-γ controls. All other wells contain cells treated with the EC20 of IFN-γ stimulation and various compounds or vehicle controls. These compound treatments elicit a variety of responses, either increasing or decreasing PD-L1 expression. Figure 7 shows a heat map from the same plate but displays the percent of cells which were viable (population 3) out of the single cell population (population 2). We expect a decrease in cell viability of about 20% to 40% in IFN-γ treated cells, compared to the non-IFN-γ treated controls. IFN-γ induces a degree of cell death in THP-1 cells due to mechanisms including activation of pro-apoptotic pathways and TNF-α signaling (Inagaki et al., 2002). These mechanisms, however, are likely distinct from the mechanism of IFN-γ induced PD-L1 expression, which is primarily moderated by the JAK-STAT pathway (Mimura et al., 2018). Furthermore, IFN-γ induced PD-L1 expression can promote tumor progression *in vivo* (Abiko et al., 2015; Galbraith et al., 2020; Qian et al., 2018). Therefore, we do not believe that the observed change in cell viability confounds this protocol’s effectiveness in identifying relevant modulators of PD-L1 expression. This is why it is important that all analysis of IFN-γ treated cells includes a viability dye as described in the protocol, and PD-L1 expression analysis is performed on viable gated cells (for example, the gate for population 3 in Figure 1). All changes in compound induced cell viability should be compared to the IFN-γ and DMSO control wells.

Ultimately, this protocol should allow for the identification of hit compounds which modulate PD-L1 expression (see Quantification and Statistical Analysis for information on hit selection). The number of hits will depend largely on the choice of compound libraries tested. Once hits are selected, they should be re-tested in dose response and with replicates. Secondary assays such as qPCR and western blotting can further confirm hits. Overall, this protocol enables flow cytometry based high throughput screening of compounds which can be managed with relatively common laboratory resources.

## Quantification and statistical analysis

Brideau and colleagues have described numerous methods to analyze high throughput screening data, discussing each method’s advantages and disadvantages (Brideau et al., 2003). Analysis will need to be tailored to the investigator’s specific needs. For this assay, we typically use the Z score statistical scoring method, where for a raw value of *x*_*i*_, the Z score is calculated by this equation:

$$z_{i}=\frac{x_{i}-\bar{x}}{S_{x}}$$

Where $\bar{x}$ is the mean of the value of all wells in a given plate excluding controls and S is the standard deviation of all wells in a given plate excluding controls (Brideau et al., 2003). For example, in a screen of about 2000 compounds for activators or inhibitors of PD-L1, we calculated Z scores for the median fluorescent intensity (MFI) of PE (the fluorophore of the PD-L1 antibody) and for the percent viable cells. We defined a hit as a compound with a PE MFI Z-score absolute value ≥ 3, and a percent viable Z score > −1. Compounds with a percent viable Z score of less than −1 were initially excluded to prevent confounding results caused by toxicity, but these compounds were noted for potential future rescreening at lower doses. In a screen with a larger number of compounds, more stringent cut offs for hits should be applied (i.e., PE MFI Z-score absolute value ≥ 4 or 5). This method is only suitable if it can be reasonably assumed that hits will be rare and that the screened compounds are functionally and structurally unrelated. Note that it is important to monitor results for bias introduced by “edge effects” or other sources (Caraus et al., 2015). Independent of the method used to flag “hit” compounds, all hits should be rescreened in replicate and in dose response before progressing with further investigation.

## Limitations

This method is designed for analysis of THP-1 cells stimulated with IFN-γ, which leaves the cells with mostly non-adherent or only loosely adherent, monocyte-like morphology. This protocol is not suitable for THP-1 cells treated with PMA or any other stimulation which might induce macrophage differentiation, as this will lead to the cells becoming tightly adherent to plates and difficult to analyze by flow cytometry.

This method may be unsuitable for analysis of large numbers of fluorophores. Larger numbers of fluorophores may require compensation to correct for spectral overlap, additional controls, and the sampling of larger numbers of cells.

## Troubleshooting

### Problem 1

Cells adhere too firmly to the bottom of plates.

### Potential solution

This may indicate that too high concentrations of IFN-γ are being used. Lower the amount of IFN-γ. If the problem persists, the cells can be washed with PBS supplemented with 1 mM EDTA to help loosen any adhesion to plates. Ultra-low attachment 384 well plates can also be employed.

### Problem 2

Low number of events per well during flow cytometry analysis.

### Potential solution

This may be caused by cells which have settled in the wells or remain partially pelleted. Vortex plates thoroughly and increase the frequency of inter-well shaking during flow cytometry analysis from every 12 wells to every 6 wells. The investigator can also decrease the volume of FACS buffer in the final resuspension of cells.

Additionally, investigators can try increasing the sample time for each well, but this tends not to work as well as decreasing the volume of the cell suspension.

### Problem 3

THP-1 cells do not express PD-L1 consistently or at high levels following IFN-γ stimulation.

### Potential solution

THP-1 cells which have been in culture for too long (“high passage”) or have been cultured at too high a density may have inconsistent response to IFN-γ. Thaw a new stock of low passage THP-1 cells. Also, consider redoing the IFN-γ titration to ensure that stocks have not lost potency. This is especially pertinent if a new batch of IFN-γ has been purchased or if the existing batch has been in storage for more than 1 year.

### Problem 4

“Edge effects” or other positional bias observed.

### Potential solution

In any high throughput screening assay, there is a risk of bias occurring due to the position of wells within a plate. This is commonly seen in the form of “edge effects”, where the edge wells of a plate will provide different responses due to differences in media evaporation, temperature changes or other factors. This can be mitigated by not using the two rows or columns on the edges of each side of a 384 well plate, and instead filling those wells with sterile PBS. Additionally, allowing the plate to incubate at approximately 20°C for 1 h after adding cells to the plate can reduce edge effects (Lundholt et al., 2003). Plates can also be sealed with gas permeable seals. Furthermore, investigators can use statistical methods to analyze data which account for positional effects, such as the B-score metric (Malo et al., 2010).

### Problem 5

High background fluorescent signal from negative control cells (without IFN-γ) or low signal from positive control cells (with IFN-γ and DMSO).

### Potential solution

Titrate the antibody. Antibodies can vary in staining efficiency. Variance can even occur in different lots from the same manufacturer. To titrate an antibody against PD-L1 or any other target, test varying concentrations of antibody against positive and negative control cells. In this case, this would be THP-1 cells either with or without IFN-γ. Determine which antibody concentration provides the greatest separation between the positive and negative control.

## Resource availability

### Lead contact

Further information and requests for resources and reagents should be directed to and will be fulfilled by the lead contact, Luke Lairson (llairson@scripps.edu).

### Materials availability

This study did not produce unique reagents.

### Data and code availability

This study did not produce new code.
